# Exploration and Strategy Analysis of Mental Health Education for Students in Sports Majors in the Era of Artificial Intelligence

**DOI:** 10.3389/fpsyg.2021.762725

**Published:** 2022-03-03

**Authors:** Liang Liang, Yong Zheng, Qiluo Ge, Fengrui Zhang

**Affiliations:** ^1^College of Sports Science, Changsha Normal University, Changsha, China; ^2^Physical Education Institute, China West Normal University, Nanchong, China; ^3^Department of Literature and Law, Nanchang Jiaotong Institute, Nanchang, China; ^4^College of Life Science, Sichuan Agricultural University, Yaan, China

**Keywords:** sports major, mental health education, big data, adaptive path, artificial intelligence

## Abstract

This study aims to explore new educational strategies suitable for the mental health education of college students. Big data and artificial intelligence (AI) are combined to evaluate the mental health education of college students in sports majors. First, the research status on the mental health education of college students is introduced. The internet of things (IoT) on mental health education, a structure based on big data and convolutional neural network (CNN), is constructed. Next, the survey design and questionnaire survey are carried out. Finally, the questionnaire data are analyzed and compared with the mental health status under traditional education. The results show that the CNN model has good accuracy and ability to distinguish symptoms, so it can be applied to the existing psychological work in colleges. In the symptom comparison survey, under the traditional education and big data network, the number of college students with mild mental health problems is found to be 158 (84.9%) and 170 (91.4%), respectively. It indicates that the number of college students with moderate mental health problems decreases significantly. In the comparative investigation of the severity of mental problems, the number of students with normal mental health, subhealth, and serious mental health problems under the background of traditional mental health education is 125 (67.2%), 56 (30.1%), and 5 (2.7%), respectively. The mental health status of college students under the influence of big data networks on mental health education is better than that of traditional mental health education. There are 140 students with normal mental health, a year-on-year increase of 16.7%. In the comparative survey of specific mental disorders, students with obsessive-compulsive symptoms under traditional mental health education account for 22.0% of the total sample, having the largest proportion. In the subhealth psychological group under the big data network on mental health education, the number of hostile students decreases by 7, which is the psychological factor with the most obvious improvement. Hence, the proposed path of mental health education is feasible.

## Introduction

Recently, the living standards of the people have been improving day by day, the stimuli encountered in personal life are complex, and the life pressure is also increasing. The accumulation of pressure has an increasingly greater impact on personal mental health. More and more mental diseases and mental disorders begin to appear in people’s lives and affect people’s normal lives. Therefore, people focus more on mental health. College students are reserved talents for national development, and their mental health problems have gradually attracted people’s attention (Fang, 2021). The college stage is the transitional stage of life, which needs to complete the transformation from school to society (Porru et al., 2021). There are many factors affecting the mental health of college students, including the learning environment, social environment, employment pressure, and so on (Tran et al., 2021). At present, in the Chinese college, about 30% of college students suffer from mental diseases to varying degrees, one-third of which has more serious mental diseases (Ochnik et al., 2021). In recent years, college students frequently drop out of school and commit suicide due to mental illness (Nottage et al., 2021). Given the current situation of ’the mental health of college students, colleges have adopted various forms of mental health education. The most commonly used education is to develop mental health education courses, mainly through setting up mental health lectures and mental health counseling (Cage et al., 2021). The purpose of this study is to explore the mental health status of physical education students.

Sports major is to cultivate all kinds of sports talents. The training content of sports majors is mainly related to the theoretical knowledge and skills of sports. The evaluation method differs from traditional art and science, focusing on experimental evaluation (Slingerland et al., 2021). Therefore, there are significant differences in mental health between students in sports majors and students majoring in traditional arts and sciences (Li and Shi, 2021). Studies have shown that the mental health status of students in sports majors is much better than that of students in other majors, but reasonable and feasible mental health education is still needed (Frank et al., 2021).

Network education mode is a new educational mode rising with the progress and development of network technology. Compared with the traditional education mode, using the network to carry out mental health education in colleges is more suitable for the development trend of modern society. Traditional mental health education has been mainly carried out offline for a long time. The communication between teachers and students is limited in multiple aspects, and students’ problems cannot be solved (Maldari et al., 2021). The era of big data has come with the rapid progress of Internet technology. Applying artificial intelligence (AI) technology to the mental health education of college students is a current research hotspot because it can greatly improve the efficiency of education (Yang, 2021). At present, the construction method of big data mental health education network is mainly to establish a database, establish a network education platform, and use the network to assist mental health tracking treatment, which has greater convenience. The development of deep learning technology provides favorable conditions for the renewal of the educational model. In particular, convolutional neural network (CNN) technology is widely used in the field of education because of its unique structure and text processing ability. Therefore, CNN technology will be used to analyze educational strategies.

Today, there are still differences in mental health education in different regions, and the achievement level of mental health education is uneven. Therefore, first, the current situation of ’the mental health education of college students is introduced based on the big data, the Internet of things (IoT) technology, and AI technology. Next, the early warning module of psychological state is designed based on CNN, and the mental health education network of college students is established. Finally, symptom checklist 90 (SCL-90) is used as a questionnaire to compare the educational effects of traditional mental health education and big data network on mental health education, and the advantages of the selected path, in order to obtain a set of universal mental health education methods. The research innovation is that SCL-90 is selected as the educational effect evaluation scale of big data systems and mental health education for the first time, which greatly simplifies the research and improves the exploration efficiency. This exploration provides a reference for the application research of IoT technology and AI technology in the field of mental health education in the era of big data. The research content provides the development direction for the mental health education of college students in the sports major in the era of big data.

## Literature Reviews

### Mental Health Intelligent System

First, the research status of mental health education of college students is introduced. The research on the mental health of college students in China starts earlier. At the end of the 20th century, some scholars pointed out that the prevention and treatment of individual psychological quality and individual-prone psychological diseases are the main contents of mental health education. Psychological quality education includes interpersonal communication and relationship education, intellectual development education, personality education, environmental adaptation education, and so on. The prevention and treatment education for individual-prone mental diseases includes relevant knowledge transfer, frustration education, and prevention and treatment of mental diseases (Grocke et al., 2021). Studies in the early 21st century believed that the main contents of mental health education should include publicizing the concept of psychological and physical health, realizing mental health through acceptance and respect for self, social communication, learning psychological adjustment, and introducing psychological counseling (Marques and Braidwood, 2021). Some studies pointed out that the content of mental health education of college students should include the outlook on life and values, the formation of self-awareness, personality cultivation, learning habits and abilities, interpersonal communication, love and sexual psychology, emotional experience and control, the requirements of facing setbacks, job hunting, and so on, which basically covers all aspects of the college life of the students, as well as psychological test and evaluation, psychological counseling, and psychotherapy (Niederkrotenthaler et al., 2021). The latest research by Kim and Hong (2021) shows that the basic contents of mental health education for Chinese college students include health concepts, self-awareness, interpersonal communication, learning, personality, emotion, frustration, sexual psychology, love, network, job selection, psychological counseling, and psychotherapy.

Next, the current situation of empirical research on the mental health of college students is introduced. Since the end of the 20th century, China has gradually strengthened the empirical investigation and research on the psychological status of college students while focusing on theoretical research (Huang et al., 2021). According to the characteristics of college students, researchers use various scales, such as Cattell’s 16 personality factor (16PF), university personality inventory (UPI), and SCL-90 to study the psychological status of college students. These studies can help researchers understand the psychological characteristics of college students in different periods (Wu et al., 2020). From the perspective of empirical research, the researchers measure the mental health status of college students of different majors with SCL-90 scale and draw the conclusion that the average score of SCL-90 factors, the average positive score (2.56–2.73 points), and the number of positive items (35.05–40.55) of college students are higher than those of normal domestic young people. Besides, the more common psychological problems among college students are compulsion, interpersonal relationships, and emotional depression. College students who meet the standard of medium severity account for more than 10% of the total subjects (Prével et al., 2021). Some experts studied the mental health level of students in two colleges of the same type. The results show that setting up compulsory courses in mental health education positively affects the mental health levels of college students. After systematically and comprehensively learning the relevant courses of mental health education, college students have a clearer understanding of themselves and a significantly higher self-evaluation. Meanwhile, their social anxiety and distress are lower than those who have not taken compulsory courses. There is no significant change in the longitudinal research on self-concept and social distress of students who do not study mental health education courses. According to this result, it is concluded that learning the content of mental health education is crucial for improving the mental health level of college students (Luan et al., 2020). Following the requirements of psychometrics, researchers design a questionnaire on the needs of mental health services of college students. The questionnaire involves 35 colleges and 5,058 college students in China. It systematically masters the information about the needs, characteristics, and current situation of ’the mental health services of college students, and concludes that contemporary college students have a high demand for mental health services (Moore et al., 2019). The latest research of Gao et al. (2021) shows that due to the strong demand of college students, interpersonal relationships, employment, and academic problems have become the most concerned content; most college students hope to get the services from the mental health education center of the school, life community psychological support center, and company psychological service center; college students often obtain mental health services by understanding popular science knowledge, receiving systematic health education, and consulting classmates and friends; the demand for mental health services of female college students is greater than that of the male college students, and the demand for mental health services of grade one and grade four college students is significantly higher than that of grade two and grade three college students (Gao et al., 2021). Yang et al. (2020) used SCL-90 to study the mental health status of college students in sports majors. The results show that college students in sports majors have higher scores on factors, such as compulsion, paranoia, interpersonal relationship, hostility, and depression (Yang et al., 2020). The latest research of Son et al. (2020) on the mental health status of college students in sports majors shows that the scores of urban college students with healthy and harmonious parents are significantly lower than those in rural areas, those who lack family members, and those who have poor family relations. Compared with the latter, the former shows a higher level of mental health. Specifically, at the personal level, the study found that the SCL-90 scores of students who are satisfied with their major, have no learning pressure or less pressure, and do not have to worry about looking for a job are significantly lower than those who are dissatisfied with their major, have learning pressure and worry about looking for a job. Love also affects the mental health of the students. In the test, the scores of lovelorn and one-sided love students on SCL-90 are significantly higher than those without these conditions. After these detailed comparative studies, the researchers found that if schools, society, families, and individuals can form a linkage mechanism and work together, it will effectively improve the mental health level of college students.

The last is the introduction of the research status of ’the mental health of college students in the era of big data. The research on mental health education abroad starts earlier, and multiple colleges have established a relatively complete mental health network education system (Grasdalsmoen et al., 2020). The developed Western countries store the correction schemes of mental diseases in the network resource database, establish the network mental health education system, and use the network for the auxiliary treatment of mental health. Meanwhile, student counseling files are established through the network to track counseling at any time (Lan, 2021). The research on the network model of mental health of college students in China is still in its infancy. The latest research of King et al. (2021) shows that some colleges have established mental health education websites and achieved good results.

It reveals that China’s research on mental health education of college students mainly focuses on the following aspects: the cultivation of college students’ good mental quality and the prevention and treatment of college students’ mental diseases, the channels and methods of publicizing health concepts and explaining practical mental health, paying attention to the cultivation of values, self-awareness and interpersonal communication of college students, and their choice problems, such as job selection, employment, love and emotion management, strong willed education, and network psychology and debugging.

Recently, scholars have also listed network psychology as the content of mental health education, showing that the research content has kept pace with the times on a relatively comprehensive basis, and provides good theoretical support for follow-up research. All aspects of the literature suggest that the mental health problems of college students have become a crucial aspect perplexing the growth of college students; college students have great demand for mental health services, so it should be focused on and explored. Different studies on the mental health status of college students in sports majors have different conclusions due to different regions.

### Foundation of Mental Health Network

The network is closely related to college students and the study and life of college students through the network. To a greater extent, the network affects every aspect of them. Online education is convenient and efficient. Based on big data, it deepens the relationship between teachers and students. During online education, the degree of freedom of teaching is increased, and students can take classes according to their own needs without time and space limitations (Zhang et al., 2020). Online teaching also allows for students to consolidate and review what they have learned anytime and anywhere without the consumption of educational resources. It can broaden the platform, enrich information resources, and improve the efficiency and predictability of education.

**FIGURE 1:**
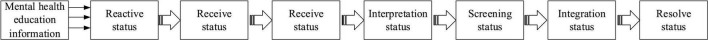
Acceptance state of big data network of ’the mental health education of college students.

The realistic basis of building college students’ mental health big data network is college students’ high acceptance of the network (Dalky and Gharaibeh, 2019). The network construction is mainly based on the theoretical method of combining moral education and mental education to cultivate psychological morality and mental health of college students, and ultimately improve their’ personalities. Figure 1 presents ’acceptance of college students regarding mental health education under the big data network.

Figure 1 reveals that the big data network acceptance state of ‘the mental health education of college students has six parts, mainly the process of mental health information being accepted in the brain of college students, including the reception, processing, and internalization of mental health information. Among them, the last two parts are the most crucial, that is, the integration and internalization of the received mental health knowledge. The key technical part is the design of the students’ mental state prediction module based on deep learning technology. This part will provide the main technical support for the construction of the whole mental health education model, which will be given in the next section.

The construction of the big data network for college students’ mental health education follows the mentioned principles. Education network needs to have certain timeliness, subjectivity, integrity, and development (Ebert et al., 2019). The main components of the big data network for college students’ mental health education include the educated, mental health information, and the receiving intermediary. The receiving intermediary is used to connect the receiving subject and the receiving object.

The contents of big data network education of ‘the mental health of college students include learning, self-awareness, employment, love, and interpersonal mental education. The related ways are to popularize psychological knowledge by opening online mental health knowledge courses. Online psychological consultation is adopted to help college students maintain their mental health. An online psychological evaluation can make teachers easily and quickly understand the mental health status of college students. College students are guided to actively explore mental health problems through the organization of an online psychological forum; college students’ online psychological mutual aid is conducted through the establishment of an online virtual community. It can help students solve their own mental health problems.

## Research Methodology and Research Model

### Construction of Deep Learning Network

**FIGURE 2:**
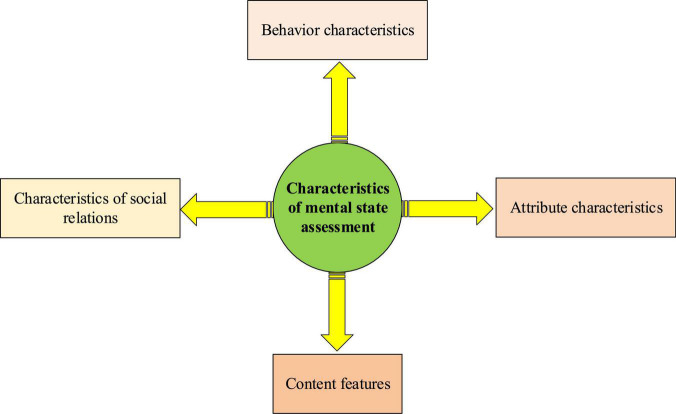
Characteristic system of mental state evaluation.

Artificial intelligence technology will be applied to mental state evaluation and early warning in the intelligent big data system. The introduction of deep learning technology will deepen the pertinence of the intelligent big data mental education system. While monitoring the students’ psychological state, it can provide personalized psychological knowledge supplements, so as to reduce the proportion of students with unhealthy psychology. The campus forum of colleges is an important place to reflect the voice of students and express their personal views. Automatic monitoring of this place is of great significance for timely mastering the current mental state of students and predicting the future dynamics of students’ psychology. The evaluation of mental health of college students based on social network’s needs to collect all kinds of characteristic indices reflecting mental health. Figure 2 is the specific content.

Figure 2 reveals that the mental state evaluation based on text content mainly focuses on four aspects: behavior characteristics, attribute characteristics, content characteristics, and social relationship characteristics.

Based on the content in Figure 2 and the school campus forum set in the research area, several sets are defined here, as shown in Equations 1–3.


(1) F={P,H,R}

(2) $$\text{P}=\left\{{\text{p}}_{1},{\text{p}}_{2},\ldots ,{\text{p}}_{\text{n}}\right\}$$

(3) $$\text{H}=\left\{{\text{h}}_{1},{\text{h}}_{2},\ldots ,{\text{h}}_{\text{L}}\right\}$$

where, *F* is the campus forum collection, *P* represents *N* different posts in the forum, *H* represents different topics of post *L*, and *R* represents the coupling relationship between posts. The mathematical definition of intelligent psychological evaluation and early warning is as follows. For any element *P* in the set *F*, a mapping relationship *m* and its corresponding set of *Q* features are searched. The expression reads as follows:


(4) $$\text{m}\,\left(\text{Q}\,\left({\text{p}}_{\text{i}},\text{H},\text{R},\text{O}\right)\right)\in \text{C}$$

*C* is the classification result from text to mental state. Equation 4 indicates that each text information published by each user corresponds to a classification. This classification can represent their mental health state, so as to remind mental health teachers in colleges to give timely intervention and personalized psychological knowledge education. Due to the unique structure of CNN, the mapping relationship *m* is used by CNN in deep learning.

**FIGURE 3:**
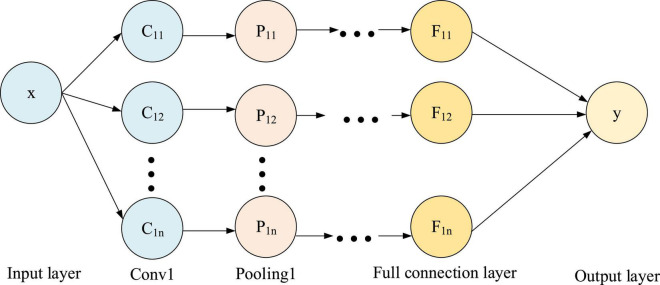
CNN structure.

Figure 3 shows the structure of CNN, which includes multiple convolution layers, pooling layers, and a fully connected layer.

The convolution layer in Figure 3 performs convolution operation, and the pooling layer performs pooling operation. In the input layer, the text content is first processed into a word vector sequence with length n with the help of linguistic inquiry and word count (LIWC), and then converted into Equations 5, 6 with the help of Word2Vec.


(5) $$\text{X}=\left[{\vec{\text{x}}}_{1},{\vec{\text{x}}}_{2},\ldots ,{\vec{\text{x}}}_{\text{i}},\ldots ,{\vec{\text{x}}}_{\text{n}}\right]$$

(6) $$\vec{{\text{x}}_{\text{i}}}\in R^{1\times d}$$

The word vector division in Equations 5, 6 can be expressed as Equation 7.


(7) $$\left\{{\text{x}}_{0:\text{h}-1},{\text{x}}_{1:\text{h}},\ldots ,{\text{x}}_{\text{i}:\text{i}+\text{h}-1},\ldots ,{\text{x}}_{\text{n}-\text{h}+1:\text{n}}\right\}$$

Convolution is a unique operation in CNN. Convolution operation can obtain local semantic information of different positions of text through convolution kernel windows of different sizes for feature detection and extraction. The partition vectors of Equation 7 are processed one by one by using the convolution operation in Equation 8.


(8) $${\vec{\text{o}}}_{\text{j}}=\partial \left(\text{f}\,\left(\text{w}\cdot {\text{X}}_{i-j+h-1}+\text{b}\right)\right)$$

where, f is the convolution kernel function used in convolution, and ${\vec{\text{o}}}_{\text{j}}$ is the eigenvalue obtained after convolution.

After the convolution is completed, the activation function is introduced to delinearize the convolution results, and then they are spliced. Equation 9 is the activation function. At this time, Equation 10 represents the characteristic matrix O output by the convolution layer.


(9) $$\text{I}\,\left(\text{x}\right)=\frac{1}{1+{\text{e}}^{-x}}$$

(10) $$\text{O}={\vec{\text{o}}}_{1}⊕{\vec{\text{o}}}_{1}+\cdots +{\vec{\text{o}}}_{n-h+1}$$

The pooling layer is used to reduce the feature dimension and prevent the reduction of operation efficiency and the fitting phenomenon caused by a too complex network. The pooling method used here is maximum pooling. The specific expression reads as follows:


(11) $$\vec{\text{O}}=\left\{\max\left({\vec{\text{o}}}_{1}\right),\max\left({\vec{\text{o}}}_{2}\right),\ldots ,\max\left({\vec{\text{o}}}_{n-h+1}\right)\right\}$$

The fully connected layer is used to connect the eigenvalues after pooling operation and convolution and take them as the final eigenvector representing text information. The equations of fully connected layer read as follows:


(12) $$\vec{\text{Q}}=\sum_{i=1}^{n-h+1}\left(\text{w}\cdot {\vec{\text{Q}}}_{\text{i}}+{\vec{\text{b}}}_{\text{i}}\right)$$

(13) $$\text{y}=\text{softmax}\,\left(\text{V}\cdot \vec{\text{Q}}+\text{b}\right)$$

(14) $$\mathrm{softmax}\,\left(x\right)=\frac{{\text{e}}^{\text{x}}}{\sum_{k=1}^{\text{K}}{\text{e}}^{\text{x}}}$$

where, Q is the original feature information obtained after full connection, and y represents the final classification result.

The text processing is based on the LIWC dictionary. In the calculation method for extracting language feature information, the post can be represented by Equation 15.


(15) $$\left\{{\text{U}}_{\text{i}}=<t_{1}^{\text{i}},t_{2}^{\text{i}},\ldots ,t_{\text{k}}^{\text{i}},\ldots ,t_{n_{\text{i}}}^{\text{i}}>\right\}$$

The frequency of occurrence of category l in dataset A can be calculated by Equations 16, 17.


(16) $$\text{TF}\,\left(l,c\right)=\frac{\sum_{i=1}^{\left|D\right|}\sum_{j=1}^{n_{\text{i}}}1_{\text{l}}\,\left({\text{t}}_{\text{j}}^{\text{i}}\right)\cdot \left({\text{u}}^{\text{i}}\right)}{\sum_{i=1}^{\text{M}}{\text{n}}_{\text{i}}\cdot 1_{\text{c}}\,\left({\text{p}}_{\text{i}}\right)}$$

(17) $$1_{\text{A}}\,\left(\text{x}\right):=\left\{ \begin{array}{cc} 1, & \mathrm{if}\,x\in A\\ 0, & \mathrm{if}\,x\notin A \end{array}\right.$$

The greater the SD of words is, the more different the words in this category are in such psychological problems. The calculation of standard deviation reads as follows:


(18) $$\delta_{\text{l}}^{2}=\sum_{\text{c}}{\left(\text{TF′(1,c)}-\mu^{2}\right)}^{2}$$

(19) $${\text{TF}}^{\prime }\,\left(\text{l},\text{c}\right)=\&\frac{\text{TF(1,c)}}{\max\left(\text{TF(1,c)}\right)}$$

(20) $$\mu \,=\frac{\sum_{c}{\mathrm{TF}}^{\prime }\,\left(1,c\right)}{\sum_{c}1}$$

In model parameter setting, it is considered that the number and proportion of samples of different categories in the dataset are quite different. The classification accuracy of the deep learning network will gradually deteriorate with the increase of the imbalance of the number of samples. Therefore, the weight of different categories of samples is distinguished. The specific method reads as follows:


(21) $${\text{w}}_{\text{c}}=\frac{1}{\text{Z}}\cdot \frac{\left|D\right|}{{\left|D\right|}_{c}}$$

(22) $$\text{Z}=\sum_{\text{c}}\frac{\left|D\right|}{{\left|D\right|}_{c}}$$

The calculation of loss function used in training reads as below:


(23) $$\text{Loss}=-\frac{1}{\text{N}}\,\sum_{\text{i}=1}^{\text{N}}\left[{\text{y}}_{\text{i}}\,\text{ln}\,\vec{{\text{y}}_{\text{i}}}+\left(1-{\text{y}}_{\text{i}}\right)\,\text{ln}\,\left(1-\vec{{\text{y}}_{\text{i}}}\right)\right]+\frac{1}{2}\,\lambda \,{||\omega ||}_{2}^{2}$$

In the determination of the number of model iterations, due to the small number of manually labeled samples, if too many iterations are carried out, the CNN network will be over fitted; if the number of iterations is too small, the accuracy of the model cannot meet the requirements. The number of model iterations and final model parameters will be given later.

The CNN model performance will be compared with the FastText model. In the experiment, the four categories previously divided are redivided into five categories. Nongreen represents a crisis, red, amber, and flagged represent nongreen classes, Urgent represents red and crisis, nonurgent represents green and amber, and all represent green, crisis, red, and amber. The evaluation indices of the model are expressed by classification accuracy (ACC) and F1. In the index performance of the model, nongreen F1 is the average value of nongreen f1, which can reflect the recognition ability of the model for all mentally unhealthy students in the validation set; Flagged F1 is the average value of Green F1, which can reflect the ability of the model to distinguish between mental health samples and mental unhealthy samples. The test results of the model will be given later.

### Questionnaire Design

To ensure the smooth progress of the research, massive documents are collected and read before the topic is put forward, such as college students’ mental health education and mental health counseling. It can help master the concept and standard of mental health. *General Psychology*, *Modern Psychology*, *Educational Statistics*, and *Principles and Methods of Sports Scientific Research* are read to master the training process, psychological experiment sampling, and statistical methods of sports specialty (Deng et al., 2020). Academic theses, such as *Chinese Mental Health Journal*, *Chinese Journal of School Doctor*, *Chinese Journal of Clinical Psychology*, *Journal of Beijing Sports University*, *Health Psychology Journal*, *China Higher Education*, *Chinese Journal of School Health*, *Health Psychology Journal*, and *Youth Children Research and Practice* and other relevant journals are read to master the current research progress in this field (Fu et al., 2021); meanwhile, relevant academic theses websites, such as the China National Knowledge Infrastructure and VIP are searched and consulted. Relevant literature is being collected and mastered to make full preparations for further research (Seehuus et al., 2021).

Symptom checklist 90 is a scale widely used by hospitals, schools, and other institutions to evaluate the psychological symptoms of the subjects (Dang et al., 2021). The reliability and validity of the scale for various symptoms are good. The test is applicable to users over the age of 16. The scale contains 10 factors and 90 items in total. The factors of the scale include somatization factor, obsessive-compulsive factor, interpersonal sensitivity factor, depression, anxiety, hostility, terror, paranoia, psychosis, and others. The items of the scale are divided into five levels according to the severity of symptoms: 1, none, 2, mild, 3, moderate, 4, relatively serious, and 5, severe, respectively, corresponding to the experience of the subjects over a period of time. The first level is subjective consciousness without symptoms; the second level is that the subjects feel that they have this symptom, but it has no actual impact on themselves; the third level is that individuals feel that they have this symptom, which has a certain impact on themselves; the fourth level is that individuals feel that this symptom occurs more often and has a considerable impact on themselves; the most serious degree is that individuals feel that the description of a symptom is very serious in the frequency and intensity of their own experience. The scores of each factor reflect the mental health level of the subjects, and there is a certain corresponding relationship. The lower the level of mental health is, the higher the score of the scale test is. The main reason for using SCL-90 is that compared with other scales, it has more test contents and can reflect richer symptom experience, including individual behavior, emotion, feeling, consciousness, thinking, interpersonal relationship, diet, sleep, and living habits. It can also accurately measure the subjects’ conscious symptoms. Based on the actual situation of more data to be collected, the number of subjects to be investigated, and the age of subjects, SCL-90 is finally selected as the questionnaire tool to evaluate the mental health state of college students in sports majors under traditional mental health education and network education. Then, the effects of mental health education under the two paths are compared.

## Experimental Design and Performance Evaluation

### Experimental Materials and Datasets

The experimental materials and dataset of this exploration are divided into two parts: CNN processing data and mental health assessment questionnaire results data.

The data of the mental state prediction and evaluation module based on CNN comes from the internal forum of college students. Therefore, to ensure the fit of application scenarios, the text information of existing public forums is selected during model simulation. The training set of the CLPsyh2017 ReachOut forum is selected, which is divided into marked and unmarked. Among them, the labeled number of test sets is 1,188, and the unmarked number is 64,567; the labeled number of training sets is 400, and the unmarked number is 91,806. In this dataset, each piece of data is composed of posting time, author, section, number of readers, and content. The structure of individual data is expressed as follows. The posting time is represented by “Time,” the forum module where the post is located is represented by “Block,” the posting author is represented by “Author,” the number of times the post is viewed is represented by “Read,” the post text content is represented by “Text,” and the number of thumb-ups of the post is represented by “Thumb-up.”

The collected datasets are labeled into four different categories. Table 1 shows the number of training sets and test sets for each category.

**TABLE 1:** Content of dataset.

Sample category	Data volume	Specific data volume
Red	185	Training set: 137
		Test set: 48
Crisis	82	Training set: 40
		Test set: 42
Green	931	Training set: 715
		Test set: 216
Amber	390	Training set: 296
		Test set: 94
Total	1,588	Training set: 1,188
		Test set: 400
Unlabeled data	157,561	Training set: 65,755
		Test set: 91,806

The SCL-90 is used as the questionnaire. It is a scale widely used in hospitals, schools, and other institutions to evaluate people’s psychological symptoms. This test is applicable to users over 16 years old. The scale was compiled by Decratis et al. in 1975. There are 58 theme versions and 35 themes simplified versions. At present, the version is composed of 90 self-assessment items that are widely used. Therefore, this test is also called SCL-90. Grace developed the latest norm for different age groups based on the commonly used version in China, and modified the fuzzy interpretation of the original version of SCL-90 into an interpretation system that is easy to understand and suitable for Chinese people (Ahorsu et al., 2020). The SCL-90 has high reliability and validity and is widely used in the fields of medicine and education. Its main contents include somatization, depression, compulsion, anxiety, and paranoia. The scale is divided into five levels, namely severe, quite severe, moderate, mild, and normal mental health (Ströhle, 2019).

In the SCL-90 scale, a factor score less than or equal to 2 indicates the normal mental state of the subjects; the factor score between 2 and 3 indicates subhealth mental state; a score greater than or equal to 2 indicates that the subject has a mental illness. A factor score greater than or equal to 3 indicates that the subject’s mental state is more serious. About 200 college students in sports majors in four comprehensive universities in Shaanxi Province are taken as the research objects. About 200 questionnaires are distributed, 190 are recovered, and 186 are valid. The mental health state of the same group of subjects after receiving traditional mental health education is compared with that of the mental health education under a big data network. The questionnaire data are analyzed by statistical product and service solution software. The distribution of symptoms, the severity of mental health, and the specific situation of mental disorders in the two educational backgrounds are compared.

### Performance Results of Neural Network

**FIGURE 4:**
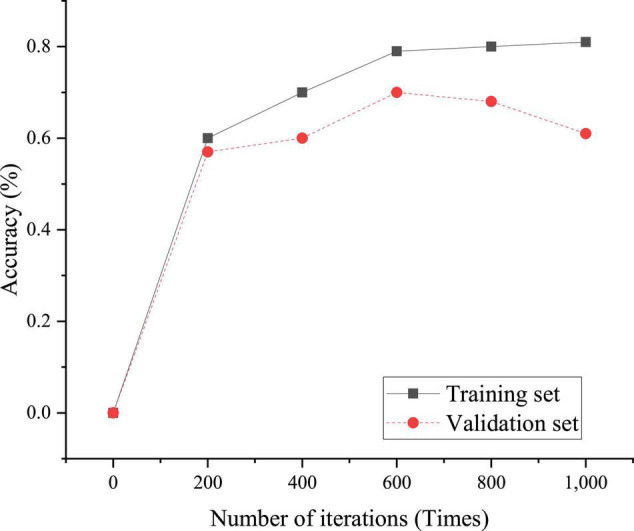
Determination of model iteration times.

Based on the previous content, the relationship between the number of iterations of CNN and the accuracy of the model is given here, as shown in Figure 4.

Figure 4 suggests that when the number of iterations of the training set is small, the accuracy consistency between the verification set and the training set is high and the model accuracy is low; when the number of iterations of the model is large, the accuracy of the training set model will increase, but the gap between the accuracy of the verification set and the training set model will become larger. At this time, the model is overfitted. Therefore, to balance the relationship between model accuracy and overfitting, the number of model iterations is 600.

Therefore, the parameter determination of the CNN model includes word vector dimension parameter value 128; the number of convolution kernels is 50; the sliding window size of convolution kernel is 2, 3, 5, and 7; the learning rate is 0.01; in the network, the dropout ratio is 0.5; the regularization coefficient of the loss function is 0.1; and the batch size is 40.

**FIGURE 5:**
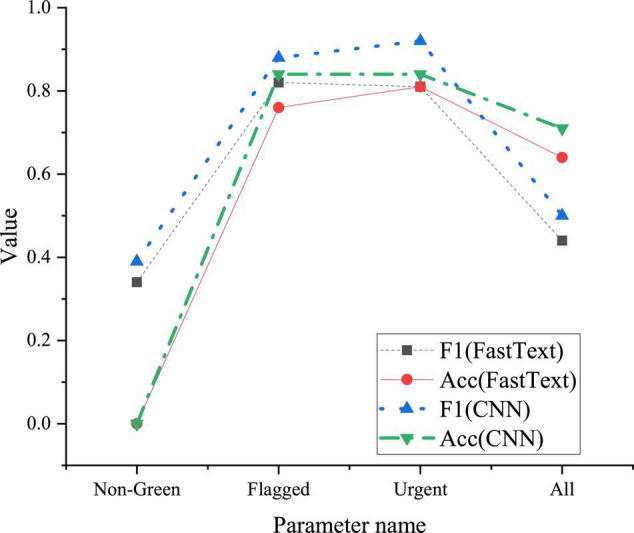
Comparison of calculation results of different models.

Figure 5 compares the calculation results of designed CNN and FastText models for different parameters.

The calculation results in Figure 5 show that in terms of the nongreen F1 index, compared with the FastText model, the CNN model improves by 0.05 and Flagged F1 improves by 0.06. This shows that the CNN model has improved in sample discrimination and recognition of nonhealthy samples. On Urgent F1, the CNN model is 0.11 higher than the FastText model. This indicator shows that the CNN model has a stronger ability to distinguish between general mental problems and urgent mental problems, and can help students quickly get help suitable for their own mental problems. From the recognition accuracy of each category of the model, the CNN model is better than the FastText model; for the accuracy of the whole sample, the accuracy of the CNN model has reached 0.71, which is higher than 0.64 of the FastText model and increased by 0.07. To sum up, the CNN model has better performance in the evaluation and early warning of mental state.

### Results of Mental Health Network

**FIGURE 6:**
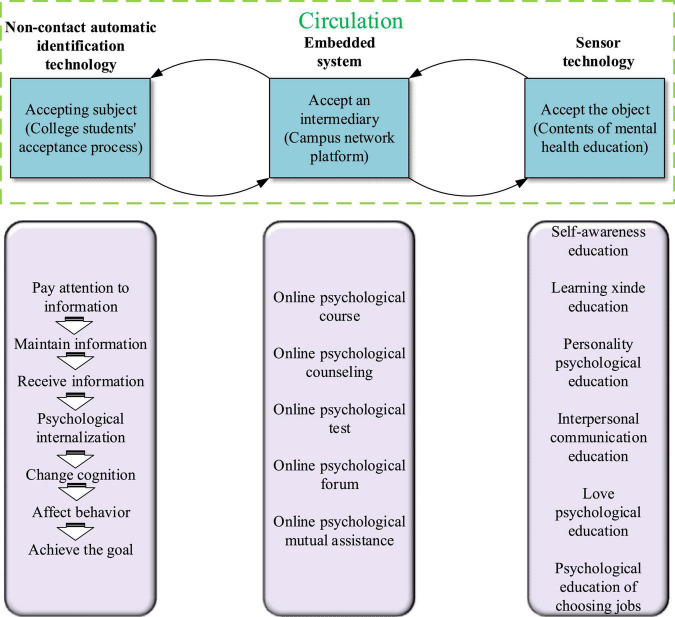
Construction structure of college students’ mental health big data network.

Based on the previous introduction, Figure 6 displays the construction structure of college students’ mental health big data network.

Figure 6 reveals that the educated, mental health information, and the receiving intermediary are in an orderly cycle and transform with each other, which together constitute the operation mechanism of college students’ mental health big data model. The information receiving process of the receiving subject is as follows: focusing on information, maintaining information, receiving information, psychological internalization, changing cognition, influencing behavior, and achieving goals. Campus network platform, as a receiving intermediary, can connect college students and mental health education information in the whole cycle.

### Comparison of Different Educational Results

Table 2 presents the grade and gender distribution in the questionnaire.

**TABLE 2:** The specific distribution of the investigated college students.

Classification	Specific	Number	Percentage
	classification	of students	(%)
Grade	Freshman	50	26.9
	Sophomore	49	26.3
	Junior	44	23.7
	Senior	43	23.1
Gender	Female	65	34.9
	Male	121	65.1

Table 2 shows that among the 186 college students who participate in the survey, there are 50 freshmen, 49 sophomores, 44 junior students, and 43 senior students, suggesting a basically uniform grade distribution. There are 65 female students (34.9%) and 121 male students (65.1%).

**FIGURE 7:**
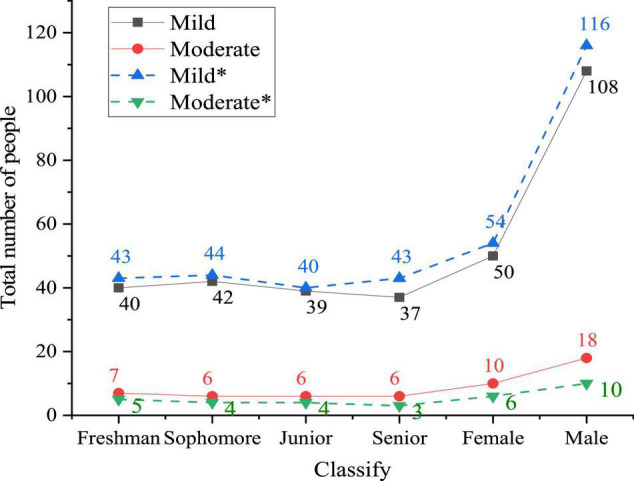
Distribution curve of symptoms of students in sports majors under two kinds of education ways (the curve with an asterisk shows the mental state of college students after receiving big data network mental education).

Under the two ways of education, first, the distribution of symptoms of students in sports majors is drawn by the scores of each factor in the questionnaire. The mental health of students is divided into mild and moderate according to the score. Figure 7 illustrates the specific situation.

Figure 7 shows that the mental state of college students under big data network mental education is significantly better than that under traditional education. The specific performance is as follows. In the college students’ mental state under traditional education, the number of students with mild mental illness is 158 (84.9%), among which 108 are male and 50 are female. About 28 students show moderate mental illness (15.1%), among which 10 are female and 18 are male. The mental state of each grade is basically the same. After mental education through the big data network, the number of college students with mild mental state increases significantly, which is 170 students (91.4%). The total number of college students with mild mental states increases by 12, with 8 males and 4 females. The number of college students with moderate mental health decreases significantly, which is 16 students, with a decrease of 12. It reveals that this mental health approach is feasible, and the reason for this change is related to the construction of college students’ campus networks. College students can solve their psychological puzzles and problems through various methods due to the increase of online psychological mutual aid and psychological forum.

**FIGURE 8:**
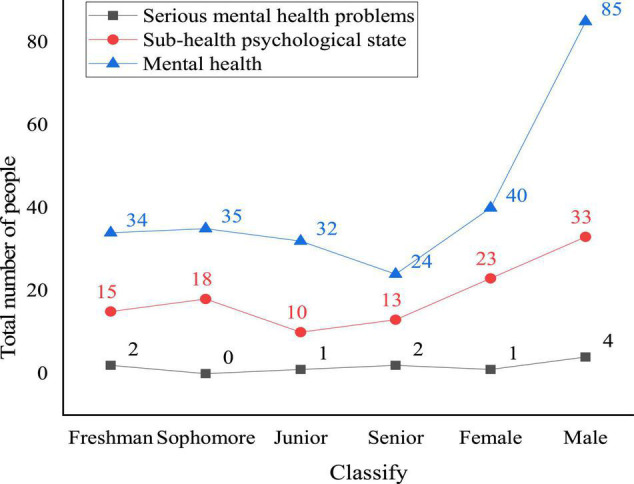
Mental health state of sports majors in comprehensive colleges in Shaanxi Province under traditional mental health education.

Then, 10 psychological factors of SCL-90 are tested. Further, the hierarchical distribution of SCL-90 and the hierarchical distribution rules of the SCL-90 scale are provided. Figure 8 displays the mental health state of college students in sports majors in Shaanxi Province under the background of traditional mental health education.

Figure 8 displays that the mental state of the investigated college students is divided into three categories: mental health, subhealth, and serious mental health problems. Under the background of traditional mental health education, the number of students with mental health is 125 (67.2%). Among them, 85 are men, accounting for 70.2% of the total number of males, and 40 are females, accounting for 61.5% of the total number of females. The number of senior students with mental health is slightly higher than that of junior students; the number of students with subhealth mental states is 56, accounting for 30.1%. Among them, female students with mental subhealth account for 35.4% of the total number of females, and male students with mental subhealth account for 27.3% of the total number of males; the number of subhealth students in each grade is basically the same. The number of students with serious mental health problems is 5, accounting for 2.7% of the total number of samples. The number of males with serious mental health problems accounts for 3.3% of the total number of males, and the number of females with the same problems accounts for 1.5% of the total number of females. There is no significant difference in the number of students with serious mental health states in different grades.

**FIGURE 9:**
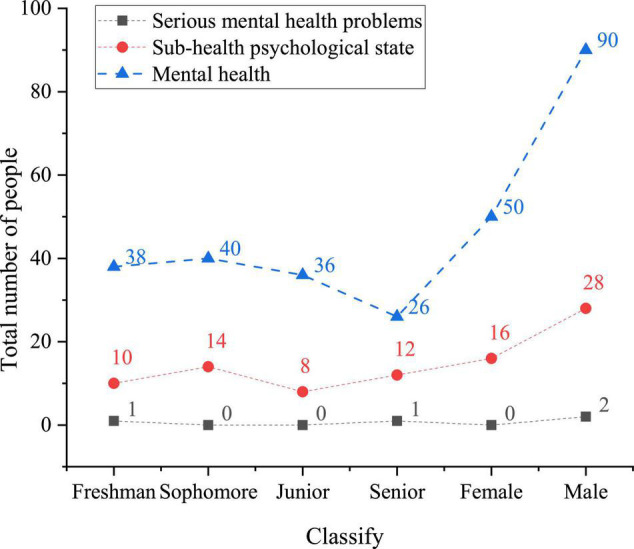
Mental health state of college students in sports majors in Shaanxi Province under the mental health education under big data network.

Figure 9 shows the mental health state of college students under the influence of big data network mental education.

Figure 9 shows that the mental health state of college students under the big data mental education network is better than that under the traditional mental health education. Among the college students who participate in network education, the number of students with normal mental health is 140, with a year-on-year increase of 16.7%. The number of the female with mental health increases by 10, and that of male increases by 5; the number of mental subhealth decreases by 12 (male 5, and female 7); the number of students with serious mental health problems decreases by 3 (male 2, and female 1). It reveals that the big data mental education network is conducive to improving the mental health of college students.

**FIGURE 10:**
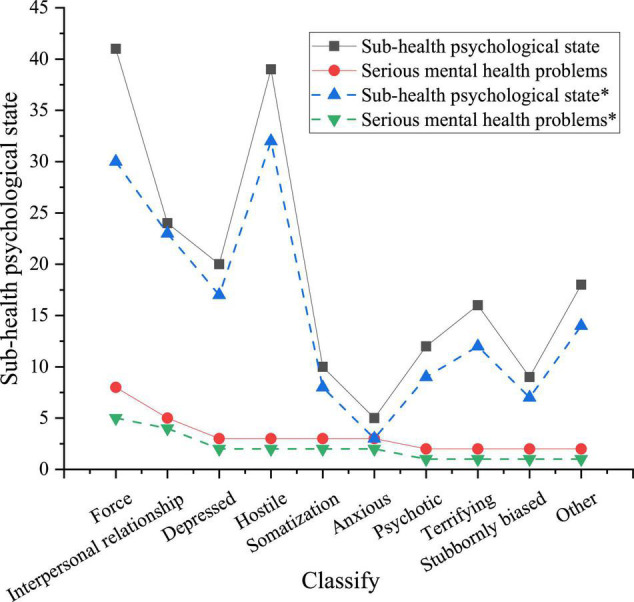
Different mental illnesses of college students in sports majors in Shaanxi Province under the background of two kinds of education.

Finally, the number of all psychological symptoms in SCL-90 is used to plot. Figure 10 presents the different mental illnesses of samples under the background of two kinds of mental health education.

Figure 10 reveals that the mental problems of college students under traditional mental health education mainly focus on compulsion, hostility, interpersonal communication, and depression. Obsessive-compulsive symptoms account for the largest proportion of students with subhealth state and serious mental health problems, which is consistent with the current Chinese mental health survey (Luan et al., 2020). Among the students with subhealth states, the top four items are obsessive-compulsive symptoms (22.0%), interpersonal problems (12.9%), depression (10.8%), and hostility (21.0%). Among the students with serious mental health problems, compulsive emotion accounts for the highest proportion (2.7%). Among the mental health problems of samples, compulsive emotion is the most serious.

## Discussion

The number of iterations of the CNN model obtained here is basically consistent with the number of iterations obtained by Kim and Kim (2021) in CNN research (Kim and Kim, 2021). It shows that applying the CNN model to mental state early warning is feasible. The CNN model can monitor the mental state in the construction of an educational network. The latest research of Yanagawa et al. (2021) on the performance of the CNN model shows that CNN has a superior prediction ability (Yanagawa et al., 2021). Figure 2 is based on the mental state early warning module established by CNN, which shows that the mental health education network system is feasible. This part will play a certain role in improving the mental health state of college students in sports majors in the study area and lay a foundation for the refinement and targeted solution of college students’ mental health problems. Connors et al. (2021) found that the mental health problems of college students can be solved immediately due to the rapid development of the network (Connors et al., 2021). The results show that under the traditional mental health education, the mental health problems of college students in sports majors have little relationship with gender. This conclusion is consistent with the conclusion of the relationship between gender and psychology drawn by Proto and Quintana-Domeque (2021). The advantages of the network in mental health education have been fully confirmed. The latest study of Černis et al. (2021) also reached this conclusion (Černis et al., 2021). The problem is expanded. Mental health education under a big data network is crucial for improving the mental state of mental subhealth groups and students with serious mental health problems. In the mental subhealth group, the number of hostile students decreases by 7, which is the psychological factor with the most obvious improvement. The reason may be the positive impact of the online psychological mutual aid system of the mental health education platform under the big data network, which relieves the hostility of college students. Therefore, the psychology of terror has been greatly improved. In general, the improvement of mental health in the mental subhealth group is more obvious than that in the serious mental problem group. The reason may be that mental subhealth people are more likely to accept mental health education under the big data network and adapt to this new education model. However, college students with serious mental health problems need mental health education and professional artificial psychological intervention. The superiority of networks in mental health education has been fully confirmed, and the latest research has also reached this conclusion. Therefore, for both the overall mental health state of students in sports majors and their specific decomposition of psychological obstacles, the effect of mental health education based on a big data network is always better than the traditional mental health education. It shows the feasibility of network education under deep learning in mental health education.

## Conclusion

Based on the college students’ mental health development under new technology, a mental health education platform for college students in sports majors based on big data and AI technology is constructed. The mental state early warning module based on CNN has good performance. On this basis, with the SCL-90 scale as a reference, the mental health state of college students under the traditional mental health education and big data network environment is compared. The results show that the main mental problems of college students under traditional mental health education are compulsion, hostility, interpersonal communication, and depression, accounting for 27%. Mental health education under IoT intelligent systems and big data are crucial for improving the mental state of mental subhealth groups and students with serious mental health problems. The number of hostile students in the mental subhealth group decreases by 7. This is the psychological factor with the most obvious improvement. The newly constructed college students’ health education platform includes three aspects: the psychological mutual aid alliance of college students in sports majors, the crisis intervention system of college students, and the construction of college students’ psychological environment. The platform has achieved good results. The mental health plan for college students is feasible. The research content has a certain reference function for future research on mental health education of college students in sports majors. There are still some research deficiencies. The psychological factors with higher scores in SCL-90 are not further analyzed. For example, the causes of obsessive-compulsive emotion among college students are not analyzed from the aspect of students’ physiological changes. Besides, the quantitative analysis of CNN application results is less, and the number of comparison models selected is not enough. Therefore, the depth of conclusions needs to be improved. In the follow-up research, the psychological factors affecting the mental health of college students in sports majors will be deeply analyzed, and the early warning results of the mental state early warning module under CNN will be quantified, so as to make the research content more complete and the conclusion more credible. This thesis will be applied to the field of mental health education and mental disease prevention in colleges to provide a reference for the development of this field.

## Data Availability Statement

The raw data supporting the conclusions of this article will be made available by the authors, without undue reservation.

## Ethics Statement

The studies involving human participants were reviewed and approved by the Ethics Committee of Changsha Normal University and China West Normal University. The patients/participants provided their written informed consent to participate in this study. Written informed consent was obtained from the individual(s) for the publication of any potentially identifiable images or data included in this article.

## Author Contributions

All authors listed have made a substantial, direct, and intellectual contribution to the work, and approved it for publication.

## Conflict of Interest

The authors declare that the research was conducted in the absence of any commercial or financial relationships that could be construed as a potential conflict of interest.

## Publisher’s Note

All claims expressed in this article are solely those of the authors and do not necessarily represent those of their affiliated organizations, or those of the publisher, the editors and the reviewers. Any product that may be evaluated in this article, or claim that may be made by its manufacturer, is not guaranteed or endorsed by the publisher.
