# Supplementary material for: Exploration and Strategy Analysis of Mental Health Education for Students in Sports Majors in the Era of Artificial Intelligence
Source: Front Psychol. 2022 Mar 3;12:762725. doi: 10.3389/fpsyg.2021.762725 (PMC8928123; doi:10.3389/fpsyg.2021.762725)
Supplement: Supplementary file 1 [file Data_Sheet_1.pdf]

Appendix 1

Questionnaire on exploration and strategy analysis of mental health education for students in sports majors in AI Era

Dear students,

Hello! Thank you for participating in this questionnaire. The questionnaire aims to realize the basic psychological status of physical education students, and finally find solutions to improve their psychological level. It is hoped that you can cooperate and fill in the questionnaire carefully. There are some questions listed in the following contents. Please read each one carefully and select one of the five options below according to your actual feelings within a week. The survey results are only for research purposes. We promise to protect your privacy. Thank you very much for your support!

Basic information

Gender:
Grades:
Age:

Symptom readme scale

Please read each of the following carefully and then choose according to the actual feelings of the last week. A, B, C, D and E represent "no, mild, moderate, relatively serious and serious" respectively.

1. Headache
A B C D E
2. Neuroticism
A B C D E
3. Unnecessary thoughts or words hover in your mind.
A B C D E
4. Dizziness or fainting
A B C D E
5. Decreased interest in the opposite sex
A B C D E
6. Demand perfection for others
A B C D E
7. Feel that others can control your thoughts
A B C D E
8. Blame others for causing trouble
A B C D E
9. Have a poor memory
A B C D E
10. Worry about your neat clothes and proper manners
A B C D E
11. Easily annoyed and excited
A B C D E
12. Thoracalgia
A B C D E
13. Fear of open places or streets
A B C D E
14. Feel your energy decline and activity slow down
A B C D E
15. Want to end your life
A B C D E
16. Hear voices that others cannot hear
A B C D E
17. Tremble
A B C D E

18. Feel that most people are untrustworthy

A	B	C	D	E

19. Poor appetite

A	B	C	D	E

20. Easy to cry

A	B	C	D	E

21. Feel shy and uncomfortable with the opposite sex

A	B	C	D	E

22. Feel cheated, trapped, or someone trying to catch you

A	B	C	D	E

23. Suddenly feel afraid for no reason

A	B	C	D	E

24. Feel like you cannot control your temper

A	B	C	D	E

25. Afraid to go out alone

A	B	C	D	E

26. Often blame yourself

A	B	C	D	E

27. Lumbago

A	B	C	D	E

28. Feel difficult to complete the task

A	B	C	D	E

29. Feel lonely

A	B	C	D	E

30. Feel depressed

A	B	C	D	E

31. Worry too much

A	B	C	D	E

32. Not interested in things

A	B	C	D	E

33. Suffer from fear

A	B	C	D	E

34. Feelings are vulnerable.

A	B	C	D	E

35. Others can know your private thoughts.

A	B	C	D	E

36. Feel that others don't understand you and don't sympathize with you

A	B	C	D	E

37. Feel that people are not friendly to you and don't like you

A	B	C	D	E

38. Work must be done slowly to ensure that it is done correctly.

A	B	C	D	E

39. Heart is beating hard

A	B	C	D	E

40. Nausea or stomach discomfort

A	B	C	D	E

41. Feel inferior to others

A	B	C	D	E

42. Muscle soreness

A	B	C	D	E

43. Feel someone watching you and talking about you

A	B	C	D	E

44. have difficulty in falling asleep

A	B	C	D	E

45. Things must be checked over and over again.

A	B	C	D	E

46. Difficult to make a decision
A B C D E
47. Afraid of tram, bus, subway or train
A B C D E
48. Expiratory dyspnea
A B C D E
49. A chill or fever
A B C D E
50. Avoid something, occasion, or activity because you are afraid
A B C D E
51. The brain is empty.
A B C D E
52. Numbness or tingling
A B C D E
53. There is a feeling of infarction in the throat.
A B C D E
54. Feel no future and hopeless
A B C D E
55. Cannot concentrate
A B C D E
56. Feel a part of the body weak
A B C D E
57. Feel nervous or easily nervous
A B C D E
58. Feel heavy on your hands or feet
A B C D E
59. Think of death
A B C D E
60. Eat too much
A B C D E
61. Feel uncomfortable when others look at you or talk about you
A B C D E
62. There are some ideas that don't belong to you.
A B C D E
63. Have the impulse to hit or hurt others
A B C D E
64. Wake up too early
A B C D E
65. You must wash your hands, count or touch something repeatedly.
A B C D E
66. Cannot sleep well
A B C D E
67. Have the urge to break or destroy something
A B C D E
68. There are some thoughts or ideas that others don't have.
A B C D E
69. Feel nervous about others.
A B C D E
70. Feel uncomfortable in places with many people such as shops or cinemas.
A B C D E
71. Feels that everything is difficult
A B C D E
72. Bursts of fear or panic
A B C D E
73. Feel uncomfortable eating in public
A B C D E

74. Often argue with others

A	B	C	D	E

75. Feel nervous when you're alone

A	B	C	D	E

76. Others didn't give you a proper evaluation of your achievements.

A	B	C	D	E

77. Feel lonely even with others

A	B	C	D	E

78. Feel restless

A	B	C	D	E

79. Feel that you are worthless

A	B	C	D	E

80. What feels familiar becomes strange or doesn't seem to be true.

A	B	C	D	E

81. Yell or drop something

A	B	C	D	E

82. Afraid to faint in public

A	B	C	D	E

83. Feel that others want to take advantage of you

A	B	C	D	E

84. Worried about some ideas about "sex"

A	B	C	D	E

85. You think you should be punished for your fault.

A	B	C	D	E

86. Feel the need to get things done quickly

A	B	C	D	E

87. Feel like you have a serious problem with your body

A	B	C	D	E

88. Never feel close to anyone else

A	B	C	D	E

89. Feel guilty

A	B	C	D	E

90. Feel something wrong with your brain

A	B	C	D
